# Editorial for the Special Issue Titled “Adenosine Metabolism: Key Targets in Cardiovascular Pharmacology”

**DOI:** 10.3390/ph17060751

**Published:** 2024-06-07

**Authors:** Barbara Kutryb-Zając

**Affiliations:** Department of Biochemistry, Medical University of Gdańsk, 80-211 Gdańsk, Poland; b.kutryb-zajac@gumed.edu.pl; Tel.: +48-58-349-14-14

Adenine nucleotides and adenosine maintain cardiovascular homeostasis, producing diverse effects by intracellular and extracellular mechanisms [1]. Intracellularly, adenosine triphosphate (ATP) is the critical energy currency molecule, mainly used to drive energy-consuming processes such as active transport, biosynthesis, and cell motility [2]. The release of intracellular ATP occurs under cellular stress conditions induced by hypoxia or inflammation via non-lytic mechanisms, including vesicular exocytosis, ion channels, and transporters, or upon cell lysis during organ injury, apoptosis, and necrosis [3]. In extracellular space, ATP is sequentially dephosphorylated to adenosine via ectonucleoside triphosphate diphosphohydrolase-1 (NTPDase1/CD39) and ecto-5′-nucleotidase/CD73 activities [4]. Further, adenosine can be irreversibly deaminated to inosine by cell-surface ecto-adenosine deaminase (eADA) [5]. Both extracellular ATP and adenosine act as powerful signaling molecules, stimulating purinergic receptors on a wide range of cell types. Adenosine acts via G protein-coupled P1 receptors, named adenosine receptors (ARs), that activate (A2A and A2B) or inhibit (A1 and A3) adenylyl cyclase [6]. In turn, ATP activates two P2 receptor subfamilies, P2X and P2Y [7]. P2X receptors are ATP-specific ligand-gated ion channels that include seven receptors (P2X1–P2X7) [8]. P2Y receptors are coupled to Gq and activate phospholipase C-β (P2Y1, P2Y2, P2Y6, and P2Y11) or coupled to Gi that inhibit adenylyl cyclase (P2Y12, P2Y13, and P2Y14) [9]. As ligands, P2Y receptors recognize ATP but also ADP, UTP, UDP, UDP-glucose, and UDP-galactose [10]. Nucleotides and adenosine are key factors in the regulation of cell differentiation, platelet aggregation, blood flow, cardiac contractility, immune responses, and neuronal signaling [11]. Recent reports highlighted the therapeutic potential of purinergic pathways’ modulation in cardiovascular diseases such as aortic valve stenosis [12] and atherosclerosis [13]. This Special Issue, entitled “Adenosine Metabolism: Key Targets in Cardiovascular Pharmacology”, aims to provide evidence about the efficacy of novel pharmaceutical and non-pharmaceutical modulation of adenine nucleotide and adenosine signaling as a valuable therapeutic approach for cardiovascular pathologies.

Cassavaugh et al. and Mierzejewska et al. (contributions 1 and 7) focused on endothelial ectonucleotidases that maintain a critical balance between vascular extracellular ATP and adenosine signaling. Cassavaugh et al. reported the induction of CD39 expression and estrogen receptor alpha levels in the presence of estradiol under hypoxic conditions in the endothelium. This upregulation increased the level of extracellular adenosine, which promoted in vitro angiogenesis. The in vivo findings of reduced purinergic responses with estrogen depletion supported these outcomes. Understanding the interactions between CD39, adenosine, and estrogen signaling will improve the knowledge of the effects of hormonal regulation on the cardiovascular system and identify potential targets for future therapeutic intervention. These data suggest novel therapeutic avenues to explore in the amelioration of post-menopausal cardiovascular disease by modulation of adenosinergic mechanisms. Mierzejewska and colleagues demonstrated the vasoprotective effects of the combination of three human genes that were introduced to a mouse model using multi-cistronic F2A technology. These included genes encoding CD39, CD73, and heme oxygenase 1 (HO-1). As previously demonstrated, the introduction of a dicistronic plasmid for the co-expression of human CD39 and CD73 in porcine endothelial cells protected against H_2_O_2_-induced cytotoxicity via enhanced production of extracellular adenosine [14]. The effects observed in hCD39/hCD73/hHO-1 transgenic mice comprised diminished levels of endothelial activation and dysfunction biomarkers as well as decreased endothelium permeability under LPS-stimulated conditions. These results were in line with recently published data on L-arginine metabolism that marked endothelial dysfunction in CD73-deficient mice, highlighting the critical role of CD73-derived adenosine for vascular protection [15].

Elgebaly et al., Ahmed et al., and Zabielska-Kaczorowska et al. (contributions 2, 4, and 5, respectively) demonstrated promising therapeutic strategies based on rebuilding the pool of intracellular nucleotides in cardiac ischemic injury. Elgebaly et al. revealed that cyclocreatine phosphate (CCrP), a potent synthetic high-energy phosphate donor that is taken up into cardiac tissue to generate ATP under conditions of limited oxygen supply [16], prevents harmful downstream events of ischemia, such as inflammation, apoptosis, remodeling, and cardiac dysfunction. Ahmed et al. provided evidence of flibanserin cardioprotective efficacy via the 5-HT2A receptor gene/5-HT/Ca pathway. These effects were tested in isoproterenol-induced myocardial infarcted rats. In turn, Zabielska-Kaczorowska et al. demonstrated the effects of exogenous adenosine infusion in Langendorff-perfused rat hearts subjected to repeated ischemic events. Their outcomes provided a considerable increase in endogenous purine catabolite production after the transient infusion of adenosine, which was associated with the increase in the cardiac adenine nucleotide pool.

Miguel-Martínez et al., Sitek et al., and Silva-Velasco et al. (contributions 3, 6, and 8, respectively) investigated the pharmacological importance of purinergic receptors in the context of blood flow regulation. Sitek at al. compared the role of endogenous adenosine and its interaction with nitric oxide (NO) and H_2_O_2_ in controlling renal and systemic circulation as well as renal excretion in rats with streptozotocin-induced diabetes. These studies revealed that diabetes exerts tonic stimulatory action of A2aR on NO bioavailability but reduces P1R involvement in the control of tissue H_2_O_2_ under normoglycemia. Miguel-Martínez et al. demonstrated that adenosine-5′-(β-thio)-diphosphate (ADPβS), a stable synthetic analogue of ADP and preferential agonist at purinergic P2Y1, P2Y12, and P2Y13, inhibited a vasodepressor sensory of calcitonin gene-related peptide in healthy pithed rats, and this phenomenon was mediated by the activation of prejunctional P2Y1 and P2Y13 receptors. In turn, Silva-Velasco et al. discovered that ADPβS decreased diastolic blood pressure via peripheral activation of P2Y1 receptors and increased systolic blood pressure in pithed rats infused with methoxamine (an α1-adrenergic agonist that restores systemic vascular tone) involving peripheral activation of P2Y1, P2Y12, and P2Y13 receptors.

As the Guest Editor, I hope that the findings included in this Special Issue will inspire further investigations in this challenging field.
